# Canine and feline foetal fluids: Volume, hormonal and biochemical characterization during pregnancy

**DOI:** 10.1002/vms3.1452

**Published:** 2024-04-24

**Authors:** Hossein Sahraei, Asghar Mogheiseh, Saeed Nazifi, Mohammad‐Reza Divar, Fatemeh Iraji

**Affiliations:** ^1^ Department of clinical Sciences, School of Veterinary Medicine Shiraz University Shiraz Fars Iran

**Keywords:** allantoic, amniotic, cat, dog, fetus

## Abstract

**Background and objectives:**

This study aimed to evaluate the volume, the concentration of steroid hormones, and biochemical composition of the foetal fluids at different gestational ages in dogs and cats.

**Methods:**

Following the ovariohysterectomy, the allantoic and amniotic fluid samples were collected from pregnant bitches and queens and were assigned to different groups according to their gestational age.

**Results:**

The canine and feline allantoic fluid volume increased during pregnancy, reached its maximum values on days 40–49 and then decreased. The canine and feline amniotic fluid volume increased steadily by the last days of pregnancy. In spite of significant changes of sex hormones in the foetal fluids, their concentration and ratios were not significantly different between male and female fetuses. The canine amniotic cortisol concentration increased until days 40–49 and decreased significantly afterwards. The maximum cortisol concentrations in the feline allantoic and amniotic fluids were observed on days 50–60 and 40–49, respectively. During the canine pregnancy, the concentrations of calcium, phosphorus, chloride, sodium, triglyceride, cholesterol, total protein, albumin and the activities of aminotransferase (AST), alkaline phosphatase (ALP), amylase and gamma‐glutamyl transferase (GGT) in the amniotic fluid were higher than the allantoic fluid. The magnesium, potassium, lactate dehydrogenase (LDH) activity, creatine and lipase were higher in the allantoic fluid. In the feline allantoic fluid, potassium, magnesium, phosphorus, creatinine, albumin and glucose concentrations and the activities of creatine kinase (CK), GGT, LDH and lipase were higher. The ALP, AST activities, sodium and calcium concentrations were higher in the amniotic fluid (*p *< 0.05).

**Conclusion:**

Volume of foetal fluids was determined in dogs and cats. Concentration of sex hormones did not different between male and female fetuses.

## Graphical abstract

The volume of allantoic fluid increased during pregnancy by day 40–50 and then decreased but the amniotic fluid increased consistently by the end of pregnancy in dogs and cats. Sex steroid hormones in foetal fluids did not consistently associate with foetal sex in dogs and cats during pregnancy. The biochemicals in amniotic and allantoic fluids changed during pregnancy in according with development of foetal organs and changing in foetal metabolism.

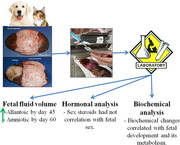

## 1 INTRODUCTION

Up to now, despite the prominent roles of foetal fluids in growth, development and physical protection, little attention has been paid to the volume and composition of these fluids. Much less is known about the source and composition of foetal fluids, but studies in humans and sheep suggest that amniotic fluid consists of secretions from the respiratory and digestive tract, urinary system, oral cavity and non‐keratinized foetal skin. In contrast, allantoic fluid is supposed to originate from allantoic membrane secretion and transportation activity during early pregnancy. As the foetal urinary system develops, the allantoic fluid becomes more similar to foetal urine (Fresno et al., 2012; Veronesi et al., 2018). Well‐known amniotic volume and composition changes in humans have made amniocentesis a practical method for foetal health assessment for the last 50 years (Underwood et al., 2005), whereas the lack of information in this field has deprived veterinarians of being aware of pathologic pregnancies and the possible special needs of abnormal newborns after birth. Analysis of bovine, ovine, equine, feline and canine foetal fluids during pregnancy made it possible to estimate the age of the fetus according to the age at which metabolic development occurs. Moreover, amniocentesis became a diagnostic method for foetal nutritional problems, detection of equine herpes virus in amniotic fluid, assess lung maturity in puppies and identifying chromosome alterations (Baetz et al., 1976; Bonte et al., 2017; Smith et al., 1997; Wales & Murdoch 1973). In 2021, two transabdominal ultrasound‐guided methods were assessed in dogs: the ‘free hand’ and the needle‐guided bracket sampling. The study concluded that foetal fluid collection is possible with relative ease and low short‐term risk, and it may pave the way for diagnostic, therapeutic and research applications in dogs. This procedure can offer valuable new insights into prenatal clinical medicine, such as diagnosing foetal deaths and early identification of heritable diseases, among others (Tal et al., 2021).

Previous studies on canine foetal fluids are limited to the investigation of the biochemical composition of foetal fluids at term (Veronesi et al., 2018) and other studies on some scattered factors of these fluids from embryonic vesicles delivered by emergency or elective caesarean section so our knowledge about the changes of biochemical components during pregnancy is rudimentary. The biochemical composition of feline foetal fluids during pregnancy has been investigated in two studies (Bigliardi et al., 2022; Fresno et al., 2012), and some differences were observed between the results of these studies. There is only one study (Wislocki, 1935) on feline foetal fluids volume; more investigations are needed in this field. A recent study examined the biochemical composition of dog foetal fluids in the latter half of pregnancy. The researchers aimed to establish the reference range for certain biochemical compositions in the foetal fluids of healthy dogs during mid‐pregnancy. Additionally, they compared the composition of amniotic and allantoic fluids across various fetuses and uteri to identify similarities and differences. Furthermore, the researchers developed an algorithm that can predict the source of the fluids by analysing the concentration of specific biochemical compounds (Tal et al., 2022). To date, no study has investigated the volume of canine foetal fluids, sex steroid hormones concentration associated with foetal sex in both feline and canine foetal fluids, and the biochemical composition changes during canine pregnancy. Therefore, this study contributes to canine and feline foetal fluids research by demonstrating volume and hormonal and biochemical changes of amniotic and allantoic fluids during pregnancy.

## 2 MATERIALS AND METHODS

### 2.1 Animal ethics statement

Experimental protocols were performed in accordance with the Iranian animal ethics framework under the supervision of the Iranian Society for the Prevention of Cruelty to Animals and University Research Council (IACUC No: 4687/63).

### 2.2 Animals and sample collection

Twenty pregnant domestic short‐hair queens and 19 pregnant mixed‐breed bitches were assigned in the present study. All animals were patients submitted to the School of Veterinary Medicine as a population‐controlling program. All animals were healthy on physical examination with no illness until the surgery. According to the gestational age, which was determined by ultrasonographic examination and measuring the gestation sac diameter (pregnancy less than 35 days) and head diameter (pregnancy greater than 35 days) (Fresno et al., 2012), bitches were divided into three groups (30–39 (*n *= 6), 40–49 (*n *= 6) and 50–60 (*n *= 7) days of pregnancy), and queens were divided into four equal groups (20–29, 30–39, 40–49 and 50–60 days). A combination of ketamine (5 mg/kg, IM), midazolam (0.2 mg/kg, IM) and buprenorphine (20 mg/kg, IM) was used as pre‐medication. Induction was performed by propofol (4 mg/kg, IV), and after intubation, anaesthesia was maintained with isoflurane (1.5%–2%) in oxygen. The ovariohysterectomy was performed by a ventral midline laparotomy and as soon as the uterus and ovaries were removed, gestation sac and head diameter were measured ultrasonographically, and the uterus was dissected to expose gestation sac. The sex of fetuses was determined macroscopically for fetuses with sexually differentiated and distinct external genital organs. A 23G needle connected to a 2‐ML syringe was used to aspirate allantoic fluid. After that, the allantoic membrane was carefully dissected, and all allantoic fluid was collected and measured by a proper syringe according to the volume of the fluids. The same procedure was performed for amniotic fluid. Eighty‐five allantoic and amniotic samples from foetal sacs were obtained, which were immediately frozen and kept at −20°C until analysis was performed within 3 months from sampling.

### 2.3 Biochemical and hormonal analyses

The activity of aspartate aminotransferase (AST), lactate dehydrogenase (LDH), creatine kinase (CK), gamma‐glutamyl transferase (GGT), alkaline phosphatase (ALP), alanine transaminase (ALT), and lipase and the concentration of creatinine, urea, amylase, triglyceride, cholesterol, albumin, glucose, magnesium, chloride, calcium and phosphorus were measured by Pars Azmoon kits (Pars Azmoon) and biochemical autoanalyser (Alpha Classic AT++; TajhizatSanjesh); sodium and potassium were measured by flame photometer apparatus (620G, Fater Electronic). Total protein was measured using Bradford's method. Solid‐phase sandwich ELISA was used to assay testosterone, oestradiol, progesterone and cortisol concentration. The kits used include testosterone ELISA kit (C.V. 4.8%; sensitivity 0.0576 ng/mL; Monokit, Monobind), oestradiol ELISA kit (C.V. 8.5%; sensitivity 8.2 pg/mL; Monokit, Monobind), progesterone ELISA kit (C.V. 7.5%; sensitivity 0.105 ng/mL; Monokit, Monobind) and cortisol ELISA kit (C.V. 7%; sensitivity 91.5 pg/mL; Monokit, Monobind). The ELISA kits were validated for dog and cat serum samples in the clinical pathology laboratory of the School of Veterinary Medicine at Shiraz University.

### 2.4 Statistical analysis

Statistical analysis was performed by GraphPad Prism 7 (GraphPad Software Inc.). The normality of data was confirmed by the Shapiro–Wilk test. The mean concentration of each hormone and biochemical factor was compared among different days of pregnancy in both amniotic and allantoic fluids. The mean concentration of mentioned factors on different days of pregnancy was compared between amniotic and allantoic fluids. The mean concentration of sexual hormones was compared between allantoic and amniotic fluids of male and female fetuses. Repeated measure two‐way ANOVA, post hoc test and *T* test were used for analysis. The significance level was determined at *p *< 0.05. Data were presented as mean ± SD.

## 3 RESULTS

### 3.1 The volume of canine foetal fluids

The allantoic fluid volume increased until days 40–50 and then decreased. Maximum volume was observed on days 40–50 compared with days 50–60 (*p *= 0.001) and days 30–40 (*p *< 0.0001). A significant difference was observed between days 30–40 and 50–60 (*p *= 0.007).

The volume of the amniotic fluid increased constantly, and the maximum volume was observed on days 50–60 in comparison to days 30–40 (*p *< 0.0001) and days 40–50 (*p *< 0.0001). A significant difference was observed between days 30–40 and 40–50 (*p *= 0.04; Table 1).

**TABLE 1:** Canine and feline amniotic and allantoic fluid volume fluctuations during pregnancy.

	Day of pregnancy			
	20–29	30–39	40–49	50–60
** Allantoic fluid volume (mL)**		15.61 ± 1.99^a^	110.12 ± 32.03^b^	66.15 ± 44.30^c^
**Dog**				
** Amniotic fluid volume (mL)**		2.32 ± 1.82^a^	12.16 ± 2.95^b^	26.8 ± 13.29^c^
** Allantoic fluid volume (mL)**	10.85 ± 2.45^b^	12.62 ± 2.4^b^	11.37 ± 0.73^b^	3.72 ± 0.83^a^
**Cat**				
** Amniotic fluid volume (mL)**	2.7 ± 0.44^b^	1.62 ± 0.89^b^	1.9 ± 0.82^b^	6.12 ± 1.47^a^

*Note*: Values are expressed as mean ± SD. Statistically significant differences (*p *< 0.05) between days of pregnancy are marked by different letters (a–c).

### 3.2 The volume of feline foetal fluids

The minimum volume of the allantoic fluid was observed on days 50–60 compared with days 20–30 (*p *< 0.0001), days 30–40 (*p *< 0.0001) and days 40–50 (*p *< 0.0001). The maximum volume of the amniotic fluid was observed on days 50–60 compared with days 20–30 (*p *< 0.0001), days 30–40 (*p *< 0.0001) and days 40–50 (*p *< 0.0001; Table 1).

### 3.3 Canine foetal fluids hormones concentration

The allantoic oestradiol concentration increased during pregnancy, and the maximum value was observed on days 50–60 compared with days 30–40 (*p *= 0.001) and days 40–50 (*p *= 0.013). Fluctuations of progesterone, testosterone and cortisol concentration were not statistically significant (Table 2).

**TABLE 2:** The concentration of biochemical and hormonal factors during pregnancy in allantoic and amniotic fluids of canine gestation sac.

	Dog					
	Allantoic fluid			Amniotic fluid		
	Day of pregnancy			Day of pregnancy		
Factor	30—39	40–49	50–60	30–39	40–49	50–60
**Glucose (mg/dL)**	32.25 ± 6.94^b^	32.49 ± 14.44^b^	13.93 ± 10.90^a^	37 ± 6.43^a^	30.66 ± 17.64^a^	24.55 ± 17.01^a^
**Urea (mg/dL)**	31 ± 7.03^a^	48.15 ± 19.76^a^	54.33 ± 23.85^a^	33.75 ± 7.28^c^	51.7 ± 12.07^b^	69.90 ± 7.28^a^
**Creatinine (mg/dL)**	0.56 ± 0.13^b^	1.85 ± 1.33^b^	6.89 ± 6.02^a^	0.56 ± 0.12^a^	0.59 ± 0.23^a^	1.12 ± 0.69^a^
**TG (mg/dL)**	13.75 ± 5.37^a^	4.07 ± 1.92^b^	4.66 ± 2.79^b^	13.05 ± 5.78^a^	8.25 ± 3.82^a^	7.27 ± 4.37^a^
**Cholesterol (mg/dL)**	1.4 ± 0.31^ab^	1.21 ± 0.73^b^	4.00 ± 3.35^a^	3.4 ± 1.61^b^	33.59 ± 18.56^a^	13.74 ± 8.27^b^
**AST (U/L)**	6.68 ± 2.84^a^	3.09 ± 2.69^b^	2.24 ± 1.82^b^	12.61 ± 9.52^a^	7.61 ± 3.95^ab^	2.94 ± 1.93^b^
**ALT (U/L)**	0.56 ± 0.45^a^	2.97 ± 2.55^a^	2.64 ± 2.29^a^	2.432.17^a^	2.00 ± 0.94^a^	1.20 ± 0.79^a^
**ALP (U/L)**	22.95 ± 13.29^a^	16.44 ± 12.71^a^	11.84 ± 7.19^a^	34.62 ± 20.31^a^	137.61 ± 71.78^b^	129.95 ± 89.39^ab^
**Ca (mg/dL)**	2.86 ± 1.89^a^	2.28 ± 1.59^a^	1.20 ± 1.17^a^	2.10 ± 0.54^a^	5.09 ± 1.67^b^	6.55 ± 2.16^b^
**P (mg/dL)**	1.98 ± 0.59^a^	1.79 ± 1.08^a^	1.83 ± 1.48^a^	3.06 ± 1.24^a^	3.88 ± 0.78^a^	4.70 ± 1.55^a^
LDH **(U/L)**	12.75 ± 11.10^a^	14.79 ± 10.50^a^	16.01 ± 15.69^a^	6.77 ± 2.43^a^	10.91 ± 5.75^a^	7.23 ± 2.93^a^
**Lipase (U/L)**	28.97 ± 18.53^a^	13.9 ± 4.23^b^	9.08 ± 4.09^b^	29.02 ± 13.98^a^	7.82 ± 3.93^b^	7.79 ± 4.15^b^
**Mg (mg/dL)**	1.71 ± 0.65^ab^	0.75 ± 0.27^a^	1.92 ± 1.35^b^	1.53 ± 0.38^a^	0.58 ± 0.29^b^	0.69 ± 0.64^b^
**GGT (U/L)**	9.05 ± 2.34^a^	12.79 ± 3.71^a^	13.10 ± 3.30^a^	6.92 ± 1.40^b^	30.01 ± 10.10^a^	12.83 ± 2.42^b^
**Amylase (U/L)**	3.60 ± 2.01^a^	24.7 ± 8.82^b^	15.85 ± 9.27^b^	4.29 ± 1.59^b^	59.2 ± 21.23^a^	18.94 ± 12.32^b^
**Cl (mmol/L)**	38.12 ± 8.98^a^	34.17 ± 8.69^ab^	25.05 ± 9.69^b^	44.37 ± 6.42^a^	51.75 ± 14.66^a^	49.13 ± 6.14^a^
**Na (mmol/L)**	117.5 ± 15.62^b^	111.3 ± 13.19^b^	66.63 ± 26.59^a^	136.75 ± 36.52^a^	109.2 ± 23.32^a^	115.90 ± 23.19^a^
**K (mmol/L)**	7.65 ± 2.17^b^	8.05 ± 1.37^b^	29.72 ± 19.24^a^	7.97 ± 1.92^a^	2.85 ± 0.70^b^	3.41 ± 1.33^b^
**CK (U/L)**	3.99 ± 2.93^a^	2.33 ± 2.23^a^	3.20 ± 2.89^a^	1.97 ± 0.92^a^	1.54 ± 1.52^a^	1.61 ± 1.58^a^
**TP (mg/dL)**	81.24 ± 2.12^b^	84.67 ± 41.49^b^	254.29 ± 162.29^a^	102.7 ± 29.8^a^	725.61 ± 309.79^b^	624.26 ± 271.00^b^
**Albumin (mg/dL)**	24.4 ± 10.64^a^	63.33 ± 51.47^a^	52.22 ± 42.06^a^	20.75 ± 0.95^b^	217 ± 127.89^a^	99.09 ± 75.02^b^
Oestradiol **(pg/mL)**	54.13 ± 12.16^b^	78.51 ± 20.28^b^	120.83 ± 42.81^a^	66.26 ± 11.18^ab^	65.44 ± 6.49^a^	73.96 ± 6.57^b^
**Progesterone (ng/mL)**	2.57 ± 1.01^a^	2.42 ± 1.89^a^	3.52 ± 1.94^a^	1.67 ± 0.26^a^	2.07 ± 0.92^a^	1.85 ± 0.82^a^
**Testosterone (ng/mL)**	0.02 ± 0.01^a^	0.03 ± 0.01^a^	0.02 ± 0.01^a^	0.02 ± 0.005^a^	0.03 ± 0.01^a^	0.03 ± 0.01^a^
**Cortisol (µg/dL)**	0.45 ± 0.1^a^	0.98 ± 0.73^a^	0.49 ± 0.27^a^	0.9 ± 0.52^ab^	1.26 ± 1.06^a^	0.36 ± 0.18^b^

*Note*: Values are expressed as mean ± SD. Statistically significant differences (*p *< 0.05) between days of pregnancy in each fluid are marked by different letters (a–c).

Abbreviations: ALP, alkaline phosphatase; ALT, alanine transaminase; AST, aminotransferase; CK, creatine kinase; GGT, gamma‐glutamyl transferase; LDH, lactate dehydrogenase.

The amniotic oestradiol concentration increased during pregnancy, and the maximum value was observed on days 50–60 compared with days 40–50 (*p *= 0.042). Cortisol concentration increased until days 40–50 and then decreased. The maximum cortisol concentration was observed on days 40–50 compared to days 50–60 (*p *= 0.02). Variations in progesterone and testosterone concentrations were not significant (Table 2).

The concentration of the allantoic oestradiol and progesterone was higher than the amniotic. A significant difference was observed on days 50–60 (*p *< 0.0001 and *p *= 0.02 for oestradiol and progesterone, respectively). Amniotic cortisol concentration was significantly higher than allantoic on days 30–40 (*p *= 0.01). No significant difference was observed between allantoic and amniotic testosterone concentration (Table 3).

**TABLE 3:** Comparison of the concentration of biochemical and hormonal factors between the allantoic and amniotic fluid of feline and canine embryonic vesicles on different days of pregnancy.

	Day of pregnancy	Dog			Day of pregnancy	Cat	
Factor		Allantoic fluid	Amniotic fluid	Factor		Allantoic fluid	Amniotic fluid
**Glucose (mg/dL)**	30–39	32.25 ± 6.94	37 ± 6.43	**Glucose (mg/dL)**	20–29	108 ± 26.66	83.12 ± 16.64
	40–49	32.49 ± 14.44	30.66 ± 17.64		30–39	15.78 ± 4.06^a^	28.87 ± 3.62^b^
	50–60	13.93 ± 10.90	24.55 ± 17.01		40–49	58.1 ± 13.14	42.1 ± 3.99
					50–60	22.07 ± 13.36	25.7 ± 11.9
**Urea (mg/dL)**	30–39	31 ± 7.03	33.75 ± 7.28	**Urea (mg/dL)**	20–29	84.7 ± 17.49	66.12 ± 17.4
	40–49	48.15 ± 19.76	51.7 ± 12.07		30–39	20.76 ± 2.87	41.37 ± 2.81
	50–60	54.33 ± 23.85	69.90 ± 7.28		40–49	95.3 ± 24.47	76.5 ± 11.42
					50–60	50.62 ± 12.69	62.12 ± 21.01
**Creatinine (mg/dL)**	30–39	0.56 ± 0.13	0.56 ± 0.12	**Creatinine (mg/dL)**	20–29	1.46 ± 0.23	0.69 ± 0.25
	40–49	1.85 ± 1.33	0.59 ± 0.23		30–39	0.80 ± 0.15	0.73 ± 0.09
	50–60	6.89 ± 6.02^a^	1.12 ± 0.69^b^		40–49	12.47 ± 7.03	0.90 ± 0.13
					50–60	54.12 ± 18.29^a^	3.81 ± 0.96^b^
**TG (mg/dL)**	30–39	13.75 ± 5.37	13.05 ± 5.78	**TG (mg/dL)**	20–29	1.98 ± 1.08	4.46 ± 3.24
	40–49	4.07 ± 1.92^a^	8.25 ± 3.82^b^		30–39	1.11 ± 0.01	2.84 ± 2.76
	50–60	4.66 ± 2.79^a^	7.27 ± 4.37^b^		40–49	7.34 ± 1.32	13 ± 6.11
					50–60	20.4 ± 16.42	8.27 ± 4.17
**Cholesterol (mg/dL)**	30–39	1.4 ± 0.31	3.4 ± 1.61	**Cholesterol (mg/dL)**	20–29	0.82 ± 0.70	2.54 ± 1.49
	40–49	1.21 ± 0.73^a^	33.59 ± 18.56^b^		30–39	0.22 ± 0.08	0.75 ± 0.51
	50–60	4.00 ± 3.35^a^	13.74 ± 8.27^b^		40–49	2.08 ± 0.87^a^	11.04 ± 1.77^b^
					50–60	8.31 ± 3.97^a^	2.24 ± 0.94^b^
**AST (U/L)**	30–39	6.68 ± 2.84^a^	12.61 ± 9.52^b^	**AST (U/L)**	20–29	1.20 ± 0.75	14.33 ± 14.00
	40–49	3.09 ± 2.69^a^	7.61 ± 3.95^b^		30–39	1.10 ± 0.57	0.80 ± 0.42
	50–60	2.24 ± 1.82	2.94 ± 1.93		40–49	13.2 ± 5.74^a^	80.7 ± 80.3^b^
					50–60	20.5 ± 7.13	8.36 ± 5.89
**ALT (U/L)**	30–39	0.56 ± 0.45	2.432.17	**ALT (U/L)**	20–29	1.79 ± 1.08	1.08 ± 0.8
	40–49	2.97 ± 2.55	2.00 ± 0.94		30–39	1.51 ± 0.62	1.54 ± 0.24
	50–60	2.64 ± 2.29	1.20 ± 0.79		40–49	1.59 ± 0.89	0.71 ± 0.69
					50–60	1.84 ± 1.02	2.56 ± 1.55
**ALP (U/L)**	30–39	22.95 ± 13.29	34.62 ± 20.31	**ALP (U/L)**	20–29	51.9 ± 26.13	70.37 ± 21.29
	40–49	16.44 ± 12.71^a^	137.61 ± 71.78^b^		30–39	26.78 ± 25.69	101.46 ± 65.75
	50–60	11.84 ± 7.19^a^	129.95 ± 89.39^b^		40–49	41.25 ± 15.52^a^	223.48 ± 95.53^b^
					50–60	122.62 ± 23.46	122.5 ± 74.42
**Ca (mg/dL)**	30–39	2.86 ± 1.89	2.10 ± 0.54	**Ca (mg/dL)**	20–29	2.83 ± 0.77	2.29 ± 0.54
	40–49	2.28 ± 1.59^a^	5.09 ± 1.67^b^		30–39	1.48 ± 0.77	2.63 ± 0.73
	50–60	1.20 ± 1.17^a^	6.55 ± 2.16^b^		40–49	2.50 ± 2.04	2.69 ± 0.91
					50–60	3.32 ± 2.35^a^	5.85 ± 2.65^b^
**P (mg/dL)**	30–39	1.98 ± 0.59	3.06 ± 1.24	**P (mg/dL)**	20–29	2.10 ± 0.98	1.30 ± 0.56
	40–49	1.79 ± 1.08^a^	3.88 ± 0.78^b^		30–39	2.11 ± 0.89	1.34 ± 0.44
	50–60	1.83 ± 1.48^a^	4.70 ± 1.55^b^		40–49	3.35 ± 0.83^a^	1.73 ± 0.33^b^
					50–60	3.74 ± 1.91	3.03 ± 1.20
**LDH (U/L)**	30–39	12.75 ± 11.10	6.77 ± 2.43	**LDH (U/L)**	20–29	25.32 ± 22.52	10.37 ± 2.68
	40–49	14.79 ± 10.50	10.91 ± 5.75		30–39	8.62 ± 0.48	7.3 ± 0.76
	50–60	16.01 ± 15.69^a^	7.23 ± 2.93^b^		40–49	44.26 ± 28.58	2.43 ± 0.76
					50–60	258.5 ± 174.22^a^	8.82 ± 0.8^b^
**Lipase (U/L)**	30–39	28.97 ± 18.53	29.02 ± 13.98	**Lipase (U/L)**	20–29	11.16 ± 4.95	19.57 ± 2.47
	40–49	13.9 ± 4.23^a^	7.82 ± 3.93^b^		30–39	11.08 ± 1.72	15.9 ± 1.3
	50–60	9.08 ± 4.09	7.79 ± 4.15		40–49	20.5 ± 8.21	14.86 ± 6.61
					50–60	43.75 ± 4.60^a^	23.22 ± 4.69^b^
**Mg (mg/dL)**	30–39	1.71 ± 0.65	1.53 ± 0.38	**Mg (mg/dL)**	20–29	0.68 ± 0.1	0.57 ± 0.32
	40–49	0.75 ± 0.27	0.58 ± 0.29		30–39	1.72 ± 0.35	1.59 ± 0.14
	50–60	1.92 ± 1.35^a^	0.69 ± 0.64^b^		40–49	2.56 ± 0.8^a^	0.85 ± 0.27^b^
					50–60	8.87 ± 1.99^a^	0.61 ± 0.31^b^
**GGT (U/L)**	30–39	9.05 ± 2.34	6.92 ± 1.40	**GGT (U/L)**	20–29	12.46 ± 2.84	19.07 ± 6.06
	40–49	12.79 ± 3.71^a^	30.01 ± 10.10^b^		30–39	10.13 ± 0.88	17.1 ± 1.91
	50–60	13.10 ± 3.30	12.83 ± 2.42		40–49	20.44 ± 7.34	21.46 ± 5.98
					50–60	26.12 ± 6.46^a^	11.50 ± 6.44^b^
**Amylase (U/L)**	30–39	3.60 ± 2.01	4.29 ± 1.59	**Amylase (U/L)**	20–29	6.15 ± 4.98	3.56 ± 0.64
	40–49	24.7 ± 8.82^a^	59.2 ± 21.23^b^		30–39	4.34 ± 1.8	7.8 ± 1.52
	50–60	15.85 ± 9.27	18.94 ± 12.32		40–49	69.34 ± 46.1^a^	14.56 ± 11.68^b^
					50–60	38.62 ± 22.9	9.93 ± 5.06
**Cl (mmol/L)**	30–39	38.12 ± 8.98	44.37 ± 6.42	**Cl (mmol/L)**	20–29	48.6 ± 11.92	46.12 ± 7.78
	40–49	34.17 ± 8.69^a^	51.75 ± 14.66^b^		30–39	16.36 ± 3.15	69.75 ± 52.19
	50–60	25.05 ± 9.69^a^	49.13 ± 6.14^b^		40–49	34.5 ± 16.88	56.2 ± 5.13
					50–60	70.37 ± 63.87	51 ± 14.12
**Na (mmol/L)**	30–39	117.5 ± 15.62	136.75 ± 36.52	**Na (mmol/L)**	20–29	126.4 ± 7.5	125.8 ± 20.87
	40–49	111.3 ± 13.19	109.2 ± 23.32		30–39	106.66 ± 13.08	120.25 ± 12.81
	50–60	66.63 ± 26.59^a^	115.90 ± 23.19^b^		40–49	94.2 ± 24.4^a^	129.4 ± 5.72^b^
					50–60	25.5 ± 14.84^a^	116.75 ± 21.54^b^
**K (mmol/L)**	30–39	7.65 ± 2.17	7.97 ± 1.92	**K (mmol/L)**	20–29	6.23 ± 1.28	7.62 ± 1.26
	40–49	8.05 ± 1.37	2.85 ± 0.70		30–39	6.06 ± 0.43	6.83 ± 0.64
	50–60	29.72 ± 19.24^a^	3.41 ± 1.33^b^		40–49	29.92 ± 15.57^a^	4.48 ± 0.44
					50–60	61.22 ± 19.93^a^	3.95 ± 0.7^b^
**CK (U/L)**	30–39	3.99 ± 2.93	1.97 ± 0.92	**CK (U/L)**	20–29	3.6 ± 2.84	2.65 ± 1.47
	40–49	2.33 ± 2.23	1.54 ± 1.52		30–39	1.36 ± 0.53	1.14 ± 0.5
	50–60	3.20 ± 2.89	1.61 ± 1.58		40–49	0.84 ± 0.29	1.14 ± 0.78
					50–60	5.15 ± 2.94^a^	0.87 ± 0.5^b^
**TP (mg/dL)**	30–39	81.24 ± 2.12	102.7 ± 29.8	**TP (mg/dL)**	20–29	449.86 ± 343.97	80.34 ± 16.53
	40–49	84.67 ± 41.49^a^	725.61 ± 309.79^b^		30–39	578.18 ± 192.82	224.15 ± 150.95
	50–60	254.29 ± 162.29^a^	624.26 ± 271.00^b^		40–49	424.09 ± 273.06	826.82 ± 160.53
					50–60	1020.66 ± 191.69	635.75 ± 529.86
**Albumin (mg/dL)**	30–39	24.4 ± 10.64	20.75 ± 0.95	**Albumin (mg/dL)**	20–29	166 ± 135.57	67.4 ± 37.66
	40–49	63.33 ± 51.47^a^	217 ± 127.89^b^		30–39	62 ± 10.95	60 ± 12.24
	50–60	52.22 ± 42.06	99.09 ± 75.02		40–49	122 ± 59.32	166 ± 28.80
					50–60	230 ± 205.79^a^	36 ± 11.40^b^
Oestradiol **(pg/mL)**	30–39	54.13 ± 12.16	66.26 ± 11.18	oestradiol **(pg/mL)**	20–29	63.60 ± 17.19	66.61 ± 16.48
	40–49	78.51 ± 20.28	65.44 ± 6.49		30–39	179.03 ± 24.04	81.05 ± 21.79
	50–60	120.83 ± 42.81^a^	73.96 ± 6.57^b^		40–49	610.70 ± 273.95^a^	219.73 ± 84.88^b^
					50–60	569.69 ± 204.86^a^	76.8 ± 9.92^b^
**Progesterone (ng/mL)**	30–39	2.57 ± 1.01	1.67 ± 0.26	**Progesterone (ng/mL)**	20–29	3.18 ± 1.82	2.37 ± 1.25
	40–49	2.42 ± 1.89	2.07 ± 0.92		30–39	31.50 ± 12.43	7.64 ± 3.38
	50–60	3.52 ± 1.94^a^	1.85 ± 0.82^b^		40–49	110.18 ± 24.51^a^	51.36 ± 23.23^b^
					50–60	45.74 ± 32.34^a^	6.26 ± 4.09^b^
**Testosterone (ng/mL)**	30–39	0.02 ± 0.01	0.02 ± 0.005	**Testosterone (ng/mL)**	20–29	0.03 ± 0.01	0.0376
	40–49	0.03 ± 0.01	0.03 ± 0.01		30–39	0.19 ± 0.1	0.04486
	50–60	0.02 ± 0.01	0.03 ± 0.01		40–49	4.30 ± 2.84^a^	0.14364^b^
					50–60	0.18 ± 0.09	0.04974
**Cortisol (µg/dL)**	30–39	0.45 ± 0.1^a^	0.9 ± 0.52^b^	**Cortisol (µg/dL)**	20–29	2.69 ± 2.08	0.44 ± 0.15
	40–49	0.98 ± 0.73	1.26 ± 1.06		30–39	7.42 ± 1.31	2.74 ± 1.43
	50–60	0.49 ± 0.27	0.36 ± 0.18		40–49	13.37 ± 13.09	26.00 ± 18.54
					50–60	22.16 ± 15.39	5.20 ± 1.83

*Note*: Values are expressed as mean ± SD. Statistically significant differences (*p* < 0.05) between days of pregnancy are marked by different letters (a–c).

Abbreviations: ALP, alkaline phosphatase; ALT, alanine transaminase; AST, aminotransferase; CK, creatine kinase; GGT, gamma‐glutamyl transferase; LDH, lactate dehydrogenase.

Despite the higher concentration of oestradiol and progesterone in the male allantoic fluid compared to the female, the difference was not significant. Despite the higher concentration of oestradiol in the male amniotic fluid and progesterone in the female amniotic fluid, these differences were not significant. No significant difference was observed between male and female allantoic and amniotic testosterone concentrations. The proportion of sex hormones in allantoic and amniotic fluids of male and female fetuses was not significantly different (Table 4).

**TABLE 4:** Comparison between male and female concentrations of sexual hormones (oestradiol, progesterone and testosterone) in canine and feline amniotic and allantoic fluids from day 40 to 60 of gestation.

	Dog				Cat			
	Allantoic fluid		Amniotic fluid		Allantoic fluid		Amniotic fluid	
Factor	Male	Female	Male	Female	Male	Female	Male	Female
Oestradiol **(pg/mL)**	106.49 ± 41.94^a^	94.29 ± 38.07^a^	70.86 ± 8.07^a^	68.85 ± 7.60 ^a^	546.88 ± 260.58^a^	683.64 ± 154.02^a^	129.84 ± 71.27^a^	208.94 ± 95.78^a^
**Progesterone (ng/mL)**	3.32 ± 2.05^a^	2.63 ± 1.08^a^	1.88 ± 0.94^a^	2.04 ± 0.80^a^	66.86 ± 46.47^a^	110.90 ± 16.84^a^	23.26 ± 22.58^a^	47.42 ± 27.51^a^
**Testosterone (ng/mL)**	0.027 ± 0.01^a^	0.03 ± 0.01^a^	0.03 ± 0.01^a^	0.03 ± 0.01 ^a^	2.33 ± 3.49^a^	2.76 ± 1.48^a^	0.08 ± 0.09^a^	0.12 ± 0.06^a^

*Note*: Values are presented as mean ± SD. Statistically significant differences (*p *< 0.05) between males and females are indicated by different letters (a–c).

### 3.4 Feline foetal fluids hormones concentration

The allantoic oestradiol, progesterone and testosterone increased until days 40–50 and then decreased. Oestradiol concentration on days 40–50 was significantly higher than on days 20–30 and 30–40 (*p *= 0.0006 and *p *= 0.005 respectively) and on days 50–60 higher than on days 20–30 and 30–40 (*p *< 0.01 respectively). Maximum progesterone concentration was observed on days 40–50 compared to days 20–30 and 30–40 (*p *≤ 0.0001) and on days 50–60 higher than days 20–30 and different with days 40–50 (*p *= 0.02 and *p *= 0.001 respectively). Maximum testosterone concentration was observed on days 40–50 compared to days 20–30 and 30–40 (*p *= 0.001), and then on days 50–60 significantly decreased on days 40–50 (*p *= 0.001). Allantoic cortisol concentration increased during the pregnancy, and the maximum concentration was observed on days 50–60 compared to days 20–30 (*p *= 0.03; Table 5).

**TABLE 5:** The concentration of biochemical and hormonal factors during pregnancy in allantoic and amniotic fluids of feline embryonic vesicles.

	Cat							
	Allantoic fluid				Amniotic fluid			
	Day of pregnancy				Day of pregnancy			
Factor	20–29	30–39	40–49	50–60	20–29	30–39	40–49	50–60
**Glucose (mg/dL)**	108 ± 26.66^c^	89.93 ± 42.15^bc^	58.1 ± 13.14^ab^	22.07 ± 13.36^a^	83.12 ± 16.64^a^	28.87 ± 3.62^b^	42.1 ± 3.99^b^	25.7 ± 11.9^b^
**Urea (mg/dL)**	84.7 ± 17.49^b^	20.76 ± 2.87^a^	95.3 ± 24.47^b^	50.62 ± 12.69^c^	66.12 ± 17.4^ab^	41.37 ± 2.81^a^	76.5 ± 11.42^b^	62.12 ± 21.01^ab^
**Creatinine (mg/dL)**	1.46 ± 0.23^b^	0.80 ± 0.15^b^	12.47 ± 7.03^a^	54.12 ± 18.29^a^	0.69 ± 0.25^b^	0.73 ± 0.09^b^	0.90 ± 0.13^b^	3.81 ± 0.96^a^
**TG (mg/dL)**	1.98 ± 1.08^b^	1.11 ± 0.01^b^	7.34 ± 1.32^ab^	20.4 ± 16.42^a^	4.46 ± 3.24^b^	2.84 ± 2.76^b^	13 ± 6.11^a^	8.27 ± 4.17^ab^
**Cholesterol (mg/dL)**	0.82 ± 0.70^b^	0.22 ± 0.08^b^	2.08 ± 0.87^b^	8.31 ± 3.97^a^	2.54 ± 1.49^b^	0.75 ± 0.51^b^	11.04 ± 1.77^a^	2.24 ± 0.94^b^
**AST (U/L)**	1.20 ± 0.75^b^	1.10 ± 0.57^b^	13.2 ± 5.74^a^	20.5 ± 7.13^a^	14.33 ± 14.0^ab^	0.80 ± 0.42^a^	80.7 ± 80.3^b^	8.36 ± 5.89^ab^
**ALT (U/L)**	1.79 ± 1.08^a^	1.51 ± 0.62^a^	1.59 ± 0.89^a^	1.84 ± 1.02^a^	1.08 ± 0.8^ab^	1.54 ± 0.24^ab^	0.71 ± 0.69^a^	2.56 ± 1.55^b^
**ALP (U/L)**	51.9 ± 26.13^b^	26.78 ± 25.69^b^	41.25 ± 15.52^b^	122.62 ± 23.46^a^	70.37 ± 21.29^b^	101.46 ± 65.75^ab^	223.48 ± 95.53^a^	122.5 ± 74.42^ab^
**Ca (mg/dL)**	2.83 ± 0.77^a^	1.48 ± 0.77^a^	2.50 ± 2.04^a^	3.32 ± 2.35^a^	2.29 ± 0.54^b^	2.63 ± 0.73^b^	2.69 ± 0.91^b^	5.85 ± 2.65^a^
**P (mg/dL)**	2.10 ± 0.98^a^	2.11 ± 0.89^a^	3.35 ± 0.83^a^	3.74 ± 1.91^a^	1.30 ± 0.56^b^	1.34 ± 0.44^b^	1.73 ± 0.33^ab^	3.03 ± 1.20^a^
**LDH (U/L)**	25.32 ± 22.52^b^	42.2 ± 23.50^b^	66.4 ± 38.71^b^	258.5 ± 174.22^a^	10.37 ± 2.68^c^	7.3 ± 0.76^b^	2.43 ± 0.76^a^	8.82 ± 0.8^bc^
**Lipase (U/L)**	11.16 ± 4.95	11.08 ± 1.72	20.5 ± 8.21	43.75 ± 4.60	19.57 ± 2.47^ab^	15.9 ± 1.3^ab^	14.86 ± 6.61^a^	23.22 ± 4.69^b^
**Mg (mg/dL)**	0.68 ± 0.1^b^	1.72 ± 0.35^b^	2.56 ± 0.8^b^	8.87 ± 1.99^a^	0.57 ± 0.32^b^	1.59 ± 0.14^a^	0.85 ± 0.27^b^	0.61 ± 0.31^b^
**GGT (U/L)**	12.46 ± 2.84^b^	10.13 ± 0.88^c^	20.44 ± 7.34^ab^	26.12 ± 6.46^a^	19.07 ± 6.06^ab^	17.1 ± 1.91^ab^	21.46 ± 5.98^a^	11.50 ± 6.44^b^
**Amylase (U/L)**	6.15 ± 4.98^b^	4.34 ± 1.8^b^	69.34 ± 46.1^a^	38.62 ± 22.9^ab^	3.56 ± 0.64^a^	7.8 ± 1.52^a^	14.56 ± 11.68^a^	9.93 ± 5.06^a^
**Cl (mmol/L)**	48.6 ± 11.92^a^	16.36 ± 3.15^a^	34.5 ± 16.88^a^	70.37 ± 63.87^a^	46.12 ± 7.78^a^	69.75 ± 52.19^a^	56.2 ± 5.13^a^	51 ± 14.12^a^
**Na (mmol/L)**	126.4 ± 7.5^c^	106.66 ± 13.08^bc^	94.2 ± 24.4^b^	25.5 ± 14.84^a^	125.8 ± 20.87^a^	120.25 ± 12.81^a^	129.4 ± 5.72^a^	116.75 ± 21.54^a^
**K (mmol/L)**	6.23 ± 1.28^c^	6.06 ± 0.43^c^	29.92 ± 15.57^b^	61.22 ± 19.93^a^	7.62 ± 1.26^a^	6.83 ± 0.64^a^	4.48 ± 0.44^b^	3.95 ± 0.7^b^
**CK (U/L)**	3.6 ± 2.84^ab^	1.36 ± 0.53^b^	0.84 ± 0.29^b^	5.15 ± 2.94^a^	2.65 ± 1.47^b^	1.14 ± 0.5^ab^	1.14 ± 0.78^ab^	0.87 ± 0.5^a^
**TP (mg/dL)**	449.86 ± 343.97^b^	578.18 ± 192.82^b^	424.09 ± 273.06^b^	1020.66 ± 191.69^a^	80.34 ± 16.53^c^	224.15 ± 150.95^bc^	826.82 ± 160.53^a^	635.75 ± 529.86^ab^
**Albumin (mg/dL)**	166 ± 135.57^a^	62 ± 10.95^a^	122 ± 59.32^a^	230 ± 205.79^a^	67.4 ± 37.66^b^	60 ± 12.24^b^	166 ± 28.80^a^	36 ± 11.40^b^
Oestradiol **(pg/mL)**	63.60 ± 17.19^b^	179.03 ± 24.04^b^	610.70 ± 273.95^a^	569.69 ± 204.86^a^	66.61 ± 16.48^b^	81.05 ± 21.79^b^	219.73 ± 84.88^a^	76.8 ± 9.92^b^
**Progesterone (ng/mL)**	3.18 ± 1.82^c^	31.50 ± 12.43^bc^	110.18 ± 24.51^a^	45.74 ± 32.34^b^	2.37 ± 1.25^b^	7.64 ± 3.38^b^	51.36 ± 23.23^a^	6.26 ± 4.09^b^
**Testosterone (ng/mL)**	0.03 ± 0.01^c^	0.19 ± 0.1^bc^	4.30 ± 2.84^a^	0.18 ± 0.09^b^	0.03 ± 0.01^b^	0.04 ± 0.03^ab^	0.14 ± 0.1^a^	0.04 ± 0.004^ab^
**Cortisol (µg/dL)**	2.69 ± 2.08^b^	7.42 ± 1.31^ab^	13.37 ± 13.09^ab^	22.16 ± 15.39^a^	0.44 ± 0.15^b^	2.74 ± 1.43^b^	26.00 ± 18.54^a^	5.20 ± 1.83^b^

*Note*: Values are expressed as mean ± SD. Statistically significant differences (*p *< 0.05) between days of pregnancy in each fluid are marked by different letters (a–c).

Abbreviations: ALP, alkaline phosphatase; ALT, alanine transaminase; CK, creatine kinase; GGT, gamma‐glutamyl transferase; LDH, lactate dehydrogenase.

Amniotic oestradiol, progesterone, testosterone and cortisol increased until days 40–50 and then decreased. Maximum oestradiol concentration was observed on days 40–50 compared to days 20–30, 30–40 and 50–60 (*p *< 0.0009). Maximum progesterone concentration was observed on days 40–50 compared to days 20–30, 30–40 and 50–60 (*p *≤ 0.0001). Maximum testosterone concentration was observed on days 40–50 compared to days 20–30 (*p *= 0.04). Maximum cortisol concentration was observed on days 40–50 compared to days 20–30, 30–40 and 50–60 (*p *< 0.01; Table 5).

Oestradiol, progesterone and testosterone concentration were higher in the allantoic than the amniotic. Oestradiol concentration significantly differed between the allantoic and amniotic fluids on days 40–50 and 50–60 (*p *< 0.001). On days 40–50 and 50–60, progesterone concentration significantly differed between the allantoic and amniotic fluids (*p *< 0.008). A significant difference in testosterone concentration was observed between the allantoic and amniotic fluids on days 40–50 (*p *= 0.0001). Despite increasing cortisol concentration during pregnancy and higher concentrations in the allantoic, the difference between the fluids was not significant (*p *> 0.05; Table 3).

Despite the higher concentration of oestradiol and progesterone in the female allantoic fluid compared to the male, the difference was not significant. Despite the higher concentration of oestradiol and progesterone in the female amniotic fluid compared to the male, the difference was not significant. No significant difference was observed between male and female allantoic and amniotic testosterone concentrations. The proportion of sex hormones in allantoic and amniotic fluids of male and female fetuses was not significantly different (Table 4).

### 3.5 Biochemical composition of canine foetal fluids

In the allantoic fluid, creatinine, concentrations of cholesterol, magnesium and potassium and total protein albumin, urea and activity of ALT, LDH and GGT increased insignificantly (*p *< 0.05). Glucose, triglyceride, AST, lipase, chloride and sodium decreased significantly (*p *< 0.05), and ALP insignificantly. The maximum amylase activity was observed on days 40–50, and fluctuation in concentration of calcium, phosphorus and creatine kinase activity was not significant (Table 2).

In the amniotic fluid, urea, calcium and total protein concentrations increased significantly (*p *< 0.05), whereas creatinine and phosphorus increased insignificantly. AST, lipase, magnesium and potassium decreased significantly (*p *< 0.05), and glucose, triglyceride, ALT and sodium decreased insignificantly. Cholesterol, ALP, GGT, amylase and albumin increased until days 40–50 and then decreased significantly (*p *< 0.05). Fluctuation in the LDH, chloride and creatine kinase concentration was not significant (Table 2).

The concentration of calcium, phosphorus, chloride, sodium, triglyceride, cholesterol, total protein, albumin, and AST, ALP, amylase and GGT activities in the amniotic fluid was higher than in the allantoic fluid (*p *< 0.05). In contrast, magnesium, potassium, LDH, creatine and lipase in the allantoic fluid were higher than in the amniotic fluid (*p *< 0.05). ALT, urea and glucose were the same between both fluids (Table 3).

### 3.6 Biochemical composition of feline foetal fluids

In the allantoic fluid, concentrations of cholesterol, creatinine, potassium, triglyceride, magnesium and total protein and activity of lipase, amylase, AST, ALP, LDH, creatine kinase and GGT increased significantly (*p *< 0.05). However, the increase in albumin was not significant. Glucose and sodium decreased significantly (*p *< 0.05), and the fluctuations in the concentration of ALT, calcium, phosphorus and chloride were not significant. A significant irregular difference was observed in urea concentration (Table 5).

In the amniotic fluid, creatinine, phosphorus and albumin increased significantly (*p *< 0.05). CK, glucose and potassium decreased significantly (*p *< 0.05). ALP, triglyceride and total protein increased until days 40–50 and then decreased, whereas LDH and ALT decreased until days 40–50 and then increased. Irregular significant fluctuation in AST, GGT activities and cholesterol, Mg and urea concentration was observed. Fluctuation in the amylase, chloride and sodium concentration was not significant (Table 5).

The potassium, magnesium, phosphorus, creatinine, albumin and glucose concentration and activity of CK, GGT, LDH, and lipase in the allantoic fluid were higher than in the amniotic fluid (*p *< 0.05). In contrast, ALP, AST, sodium and calcium in amniotic fluid were higher than in the allantoic fluid (*p *< 0.05). ALT, chloride, triglyceride, total protein and urea were the same between both fluids. On days 40–50, the cholesterol concentration was higher in amniotic and on days 50–60 in allantoic (Table 3).

## 4 DISCUSSION

### 4.1 Volume of foetal fluids

To the author's knowledge, despite the various investigations on other mammalian foetal fluids volume, very little is currently known about feline foetal fluids volume. There is no literature about canine foetal fluids volume during gestation periods. In the study of Wislocki (1935), the volume of feline amniotic fluid increased slightly towards the middle of pregnancy (10–15 mL). After that, a scatter decline was observed, followed by an increase until the end of gestation. In comparison, the volume of allantoic fluids increased dramatically and reached higher amounts than amniotic fluid at mid‐pregnancy (20 mL) and then decreased noticeably to about 6 mL towards the term. In the current study, some decrease was observed after mid‐gestation in the volume of feline amniotic fluid, and a significant dramatic increase happened towards the end of pregnancy. The volume of feline allantoic fluid increased and reached higher amounts than amniotic fluids at mid‐gestation, after which a significant drastic decline happened until the end. Canine allantoic fluid volume increased until mid‐gestation, and the highest value was observed at this stage and, after that, significantly declined. In contrast, the amniotic fluid volume increased constantly during the pregnancy but never exceeded the allantoic volume. Mesonephros (second transient kidney), metanephros (permanent kidney) and kidney secretion make allantoic fluid. The allantoic fluid is more similar to urine due to the function of the definite kidney in late pregnancy. Urine transfers to amniotic fluid regarding urachus duct occlusion and urethra formation (Bigliardi et al., 2022). This can explain the decrease of allantoic fluid volume and the increase of amniotic fluid at the end of gestation.

### 4.2 Cortisol

The maximum cortisol concentration in the feline allantoic and amniotic fluid was observed on days 50–60 and 40–50, respectively. Fluctuations of cortisol concentration in the canine allantoic fluid were not significant. The maximum cortisol concentration was observed, similar to feline amniotic fluid, on days 40–50 of gestation. Both the fetus and the mother contribute to the composition of amniotic fluid, so maternal and foetal stress and discomfort in situations where parturition is associated with problems and even during the last days of gestation, the lack of uterus space due to the maximum body size of the fetuses can cause foetal stress, secretion of adrenocorticotropic hormone and increase in the concentration of foetal cortisol and other corticosteroids (Fresno et al., 2012; Fusi et al., 2021). For this reason, the amniotic cortisol concentration of puppies delivered by emergency c‐section was significantly higher than ones delivered by elective c‐section (10.7 ± 4.15 ng/mL vs. 5.85 ± 2.96 ng/mL), and puppies with lower amniotic cortisol concentration had higher Apgar's score. So, measuring amniotic cortisol concentration can be a valuable tool for detecting puppies who need special monitoring and observation in the early hours of life (Fusi et al., 2021). Cortisol concentration was higher in allantoic fluid (6.7 ng/mL) than the amniotic fluid (3.7 ng/mL) in small‐breed puppies delivered by elective c‐section. Puppies who did not survive the first day of life had significantly higher allantoic cortisol concentrations (Bolis et al., 2017). The cortisol concentration in the feline allantoic fluid increased significantly on day 60 of gestation, contributing to foetal cortisol secretion due to reduced space and tissue hypoxia, similar to what was seen in this study (Fresno et al., 2012). On the day of delivery, maternal plasma cortisol concentration increased significantly, returning to its base values after the last puppy's birth (Hoffmann et al., 1994).

### 4.3 Sex hormones

In canine amniotic and allantoic fluids, the concentration of oestradiol significantly increased by the end of pregnancy. Additionally, the concentration of oestradiol in allantoic fluid was significantly higher than in amniotic fluid. Similarly, in feline amniotic and allantoic fluids, the concentrations of oestradiol, progesterone and testosterone significantly increased by the end of pregnancy. Furthermore, the concentrations of oestradiol, progesterone and testosterone in allantoic fluid were higher than in amniotic fluid. In spite of significant changes of sex hormones in the foetal fluids, their concentration and ratios was not significantly different between male and female fetuses. Among domestic animals, dogs are the only ones without placental steroidogenesis activity; therefore, considering that both pregnant and non‐pregnant dogs enter dioestrus, the ovaries are the only source of steroid hormones in the blood of pregnant and non‐pregnant dogs. The concentration of progesterone increases before ovulation and luteinizes the follicle wall. At ovulation, maternal plasma progesterone concentration is at least 5 ng/mL reaching its maximum amounts (about 30 ng/mL) in the middle of pregnancy (Taverne & Noakes, 2019). The corpus luteum activity then, along with luteolysis, the concentration of progesterone decreases. During the rest of the pregnancy, it is maintained above 1.5 ng/mL until 12–24 h before delivery that a drastic decline occurs as a signal for parturition. Canine pregnancy depends on maintaining progesterone concentration, and as the placenta does not produce any steroid hormone, the corpus luteum maintains pregnancy as the only source of progesterone (Hoffmann et al., 1994; Kowalewski et al., 2021; Taverne & Noakes, 2019). Oestradiol concentration increases rapidly in pro‐oestrus. It reaches its maximum value (about 60 pg/mL) before the onset of oestrus or, on average, 1 day before the LH surge, in associated with an increase in the concentration of androstenedione and testosterone. During oestrus, it decreases sharply and becomes lower than its values at the beginning of pro‐oestrus, and after that increases parallel to progesterone during dioestrus, reaching its highest value in this stage along with the peak of progesterone, and then decreases with a gentle slope (Taverne & Noakes, 2019). The concentration of 17‐β oestradiol after mating and pregnancy has been reported to be 8–24 pg/mL with no changes during pregnancy except its decline along with progesterone before parturition, which suggests that the corpus luteum is the source of low oestrogen amounts during pregnancy, and the placenta is not involved (Hoffmann et al., 1994). The similarity of changes in the concentration of oestradiol and progesterone in the dioestrus phase and before delivery indicates the same source of secretion of these two hormones (ovaries or corpus luteum). Testicular differentiation and testosterone secretion by Leydig cells occur on day 36 of pregnancy. At the same time, the hypothalamic‐pituitary‐gonadal axis begins to develop when approximately 70% (42 days) of pregnancy has passed such a foetal masculinization, and increase of the foetal plasma androgen concentration is independent of pituitary gonadotropins. Leydig cells respond to LH from the very early stages of pregnancy (O'Shaughnessy & Fowler, 2011). The hormonal activity of foetal ovaries, production of adrenal androgens and aromatase activity of different foetal tissues are not well known, but probably, according to the late activation of the hypothalamic‐pituitary‐gonadal axis except for testes, other gonads do not have many opportunities to produce hormones and influence the hormonal composition of foetal fluids. Considering that the placenta does not play a role in the secretion of steroid hormones, the origin of the sex hormones in the foetal fluids is from the maternal blood or foetal secretions. Hormonal changes of sheep foetal fluids during the sexual differentiation period showed that the concentration of testosterone was similar in the allantoic and amniotic fluid of male, female and undifferentiated sheep fetuses, the concentration of oestradiol in allantoic and amniotic fluids of female fetuses was higher than male foetuses, as well as the concentration of oestradiol in the allantoic fluid of female fetuses was higher than undifferentiated fetuses. The progesterone concentration in the amniotic fluid of female fetuses was higher than undifferentiated ones. The progesterone concentration in the allantoic fluid after day 55 (when sexual differentiation is complete and the placenta begins to secrete steroid hormones) is significantly higher than the previous days (Sabetghadam et al., 2018). There is no difference in maternal serum testosterone concentration in pregnancies with girls or boys. However, testosterone concentration in male foetal serum, umbilical blood and amniotic fluid is higher than in female fetuses. This testosterone either cannot pass through the placenta and enter the maternal blood or is converted to oestrogen by placental aromatase (Attia et al., 2021). In other human studies, the concentration of testosterone and androstenedione in the amniotic fluid of male fetuses is higher than that of females. The concentration of oestradiol in female fetuses is higher than that of males. In addition, the sex of the fetus did not affect the concentration of oestrogen in the maternal blood (Kuijper et al., 2013; Van De Beek et al., 2004; van de Beek et al., 2009).

### 4.4 Biochemical components

In the present study, ALT activity in canine foetal fluids was similar to the results reported by Veronesi et al. (2018) on days 50–60, but no significant difference was observed between the two fluids. ALT activity showed no significant change in the allantoic fluid of cats, similar to Bigliardi et al. (2022), but in the amniotic fluid decreased until days 40–50 and after that increased. AST activity increased in the feline allantoic fluid, especially after day 45 (Bigliardi et al., 2022), and similarly, in the current study, AST activity increased significantly in the allantoic fluid of cats. In contrast, AST activity decreased in both canine foetal fluids and despite (Veronesi et al., 2018) in amniotic fluid was higher than allantoic. LDH activity in our study and Veronesi et al. (2018) was higher in the allantoic fluid. Like Bigliardi et al. (2022) and Fresno et al. (2012), LDH activity was higher in the allantoic, decreased in the amniotic fluid until days 40–50 and then increased. Very low activity of AST and especially ALT (with hepatic origin mainly) in the canine foetal fluids compared to puppies (Rørtveit et al., 2015) and adults (Dunlop et al., 2011) is probably due to the immature foetal liver. A higher activity of LDH in the allantoic fluid indicates the gastrointestinal role in the final composition of the allantoic fluid because, according to Veronesi's study, total bilirubin concentration is also higher in allantoic (Veronesi et al., 2018). ALP activity increased in the amniotic fluid of cats until days 40–50 and was higher in the amniotic fluid in both dogs and cats, similar to what was reported previously (Bigliardi et al., 2022; Fresno et al., 2012; Veronesi et al., 2018). As ALP enzyme is produced by different organs such as the kidney, liver, intestinal mucosa and placenta (Hoffmann & Solter, 2008), its higher activity in amniotic fluid can be due to the role of different foetal organs in the formation and composition of amniotic fluid and the high foetal cell proliferation activity (Veronesi et al., 2018). Increased activity of ALP in the last days of pregnancy in the amniotic fluids coincides with the period when ossification occurs. This increase is probably due to bone‐derived ALP activity in young animals too (Hoffmann & Solter, 2008). The maximum activity of creatine kinase in the feline allantoic fluid was observed on days 50–60, probably indicating the muscular development of the fetus during the end of pregnancy. Maximum creatine kinase activity in the feline allantoic fluid was observed on days 50–60, probably related to the muscle growth of the fetus, whereas, in the amniotic fluid, its activity decreased during pregnancy despite consistently similar activities during the gestation period in other studies (Bigliardi et al., 2022; Fresno et al., 2012). Creatine kinase activity in dog^’^s allantoic and amniotic fluids did not change during pregnancy, and no significant difference was observed between fluids. The reference intervals of CK for dog and cat are 52–368 and 69–214 U/L (Latimer, 2011). CK values in feline foetal fluids were lower than maternal serum values, its activity did not change during pregnancy, and no difference was observed between two fluids (Bigliardi et al., 2022; Fresno et al., 2012). CK values of canine foetal fluids at term were similar to those reported by Fresno and lower than puppies and adults’ serum values but CK activity in allantoic was higher than amniotic. Negative correlation was observed between CK activity and maternal parity (Veronesi et al., 2018); on the other hand, no difference was observed between allantoic and amniotic CK activity during the second half of pregnancy (Tal et al., 2022).

Creatinine concentration increased in the foetal fluids of both dogs and cats, similar to the results of (Bigliardi et al., 2022) but was higher in the allantoic fluid. Changes in the urea concentration in feline samples were not significant, but the maximum concentration was observed in the amniotic fluid of dogs on days 50–60. Moreover, no significant difference was observed between the two fluids in both samples of dogs and cats. Because creatinine is not absorbed into maternal blood, it accumulates in the foetal fluids (Bigliardi et al., 2022), which is likely the reason for the higher concentration in foetal fluids compared to the serum of puppies and adults (Dunlop et al., 2011; Rørtveit et al., 2015). Despite (Veronesi et al., 2018) the urea concentration being similar between the two fluids, it seems that urea accumulates in both foetal fluids in a similar pattern and is balanced by a mechanism between the two fluids, similar to the results seen in the studies of Bigliardi et al. (2022) and Fresno et al. (2012).

Amylase activity in dogs^,^ allantoic and amniotic fluids increased until days 40–50 and then decreased, but lipase decreased constantly. Lipase and amylase activity in the canine foetal fluids are much lower than in the serum of puppies and adults, suggesting little pancreatic activity during foetal development and little digestion in the developing gastrointestinal tract (Veronesi et al., 2018). Amylase is filtered by the renal glomeruli and is reabsorbed and inactivated by the tubules, so only minimal activity of amylase can be detected in the urine; also, Kupffer cells in the liver reabsorb and inactivate small activity of amylase (Latimer, 2011). Considering the role of the kidney and liver in removing, reabsorbing and deactivating amylase and lipase, the activities of these two enzymes decrease as the fetus grows and the liver and kidneys develop.

As an intracellular enzyme, GGT is involved in the movement of amino acids across cell membranes and is found in the epithelium of the bile duct, kidney, pancreas and, to a lesser extent, other organs. Foetal growth, amino acid consumption and protein production or bilirubin accumulation in foetal membranes increase this enzyme^’^s activity in foetal fluids (Bigliardi et al., 2022; Center, 2007). Assuming this, GGT activity in foetal fluids increased in this study.

Total protein, albumin, glucose, triglyceride and cholesterol as nutritional components are present in foetal fluids in lesser concentrations than maternal blood and the serum of puppies and adults, reflecting the fact that maternal blood supplies nutritional components to the fetus and foetal fluids are not an important source for foetal nutrition (Bigliardi et al., 2022). Glucose concentration in the amniotic fluid of puppies who did not survive the first day of life was significantly lower than those who did. In humans, a lower glucose concentration in amniotic is associated with intrauterine growth restriction, so measuring amniotic glucose concentration can be a valuable method to detect puppies at risk of death in the first 24 h of life (Bolis et al., 2018).

The concentration of sodium and chloride in canine and feline allantoic fluids on days 50–60 decreased. In contrast, the concentration of potassium and magnesium increased, reflecting renal maturation, high reabsorption ability and Na/K ATPase activity in the last days of gestation (Fresno et al., 2012). In the same way, human foetal kidneys can reabsorb 85%–95% of filtered sodium (Fresno et al., 2012; Oliveira et al., 2002).

## 5 CONCLUSION

Foetal growth and metabolic development were coordinated with the biochemical profiles of amniotic and allantoic fluids during pregnancy in dogs and cats. The volume of foetal fluids was determined in dogs and cats. Sex steroid hormones in foetal fluids did not consistently associate with foetal sex in dogs and cats during pregnancy.

## AUTHOR CONTRIBUTIONS

All authors contributed in all parts of study from designing study to writing and preparing manuscript. Asghar Mogheiseh, Hossein Sahraei and Saeed Nazifi contributed in study design, performing study, sampling, data collection and analysis and preparing manuscript. Mohammad‐Reza Divar and Fatemeh Iraji aimed in performing laboratory assays and study.

## CONFLICT OF INTEREST STATEMENT

The authors have no conflicts of interest to declare.

## ETHICS STATEMENT

Experimental protocols were performed in accordance with the Iranian animal ethics framework under the supervision of the Iranian Society for the Prevention of Cruelty to Animals and Shiraz University Research Council (IACUC no: 4687/63). The recommendations of European Council Directive (2010/63/EU) of 22 September 2010, regarding the standards in the protection of animals used for experimental purposes, were also followed.

### PEER REVIEW

The peer review history for this article is available at https://publons.com/publon/10.1002/vms3.1452.

## Data Availability

The corresponding author's data supporting this study^’^s findings are available upon reasonable request.
